# Development of an Easy-to-Fabricate Microdevice for Three-Dimensional Culture and Its Application to Glomerular Endothelial Cell Culture

**DOI:** 10.3390/mi16030324

**Published:** 2025-03-11

**Authors:** Miyu Yamazaki, Yasuko Kobayashi, Kiichi Sato

**Affiliations:** 1 Graduate School of Science and Technology, Gunma University, Tenjin-cho, Kiryu 376-8515, Gunma, Japan; t242a009@gunma-u.ac.jp; 2 Graduate School of Medicine, Gunma University, Showa-machi, Maebashi 371-8511, Gunma, Japan; kobayasu@gunma-u.ac.jp

**Keywords:** three-dimensional culture, hydrogel, glomerular endothelial cell, organ-on-a-chip, microphysiological system

## Abstract

The development of an organ-on-a-chip to reproduce organ functions requires the incorporation of a vascular network within the tissue to transport the necessary nutrients. Tissues thicker than 200 µm cannot survive without a capillary network, necessitating the construction of a vascular network exceeding that thickness. Therefore, we focused on the development of an inexpensive and easy-to-fabricate device for thick three-dimensional(3D)-cultured tissues. This device does not have a conventional pillar array structure, and the nutrient supply to the cells from adjacent media channels is not obstructed. Additionally, this device does not require expensive soft lithography equipment or a high-precision 3D printer to fabricate the mold. Human glomerular endothelial cells and human dermal fibroblasts were co-cultured using this device, and a 3D network of vascular endothelial cells (200 µm thick) was successfully constructed. The results of this study are expected to contribute not only to the study of angiogenesis, but also to the development of 3D tissue models that require the incorporation of capillary networks as well as the development of vascularized organ-on-a-chip and disease models for drug screening.

## 1. Introduction

Blood vessels play an important role in the transport of nutrients and waste products, communication and interactions between organs, and homeostasis [1]. The construction of microvascular structures in vitro is a primary focus in understanding diseases such as vascular malformations and disorders [2]. The development of an organ-on-a-chip technique, which reproduces the function of organs, requires capillary networks in the artificial tissue to enable nutrient transport in tissues that are too thick to be maintained by diffusion alone [3]. Tissues > 200 µm in thickness cannot survive without microvasculature [4], yet most angiogenic micromodels reported to date are thin vascular tissues of approximately 100 µm. Therefore, we focused on the development of a new microfluidic device for a three-dimensional (3D) culture that can be easily fabricated to construct a vascular network ~200 µm in thickness.

The predesign method has been reported to form functional vascular structures using prepatterned scaffolds [5,6]. However, artificial blood vessels formed using this method are considered to have poor physiological functions because they ignore the in vivo mechanism of vasculature and are different in size as well. A method utilizing spontaneous lumen formation by the self-organization of endothelial cells has been reported to combat this issue [7,8,9].

Huang et al. reported a device in which hydrogel channels for cell culture and adjacent medium channels are separated by pillar arrays as a method for constructing 3D vascular tissue models by self-organization [10]. The surface tension created by the pillars between the gel and medium channels leads to the retention of only extracellular matrix hydrogels, such as type I collagen-, Matrigel-, and fibrin gel-containing cells in the gel channel. Nutrients in the culture medium were supplied to the cells in the hydrogel through gaps between the pillars. The development of this device has enabled 3D cell culture in hydrogels. Kim et al. used this structure to construct a 3D vascular network in 2013 [11]. The device has five channels separated by pillar arrays where stromal cells were seeded in the two outer channels and endothelial cells in the central channel, with the medium introduced into the channels between them and cultured, resulting in the successful formation of a 3D vascular network. However, the depth of the channels in which the vascular network was formed was 100 µm.

This pillar array structure prevented cells and the medium outside the hydrogel from interacting with the cells inside the gel. Trietsch et al. developed a device to solve this problem where the gel channel for cell culture and the adjacent medium channel are compartmentalized by a 30 µm high phase guide [12]. Bartra et al. successfully constructed a vascular network using this device in 2022. This network spontaneously elongated through the hydrogel from one channel to another by culturing microvascular endothelial cells in one medium channel and introducing a vascular endothelial growth factor (VEGF) in the other channel [13].

The fabrication of these microdevices for 3D tissue culture requires expensive molds for injection molding, molds fabricated by soft lithography, or high-precision 3D printers [14,15,16,17,18]. Although commercial microdevices have become available in recent years, custom-made devices are expensive, and it is difficult for cell biologists to conduct experiments to develop microvascular models [19].

Therefore, we aimed to develop a fabrication method for 3D culture microdevices that can easily fabricate molds by combining plastic strips and an acrylic plate, which are used as materials for hobby micromodel crafting, such as plastic models. The advantages of using inexpensive and commercially available plastic strips, over the photolithography method are that they do not require complicated fabrication processes, are simple and inexpensive, and do not require special equipment for fabrication. When glomerular endothelial cells were cultured using a self-made microdevice fabricated using this mold, a 3D endothelial network with a thickness of 200 µm was constructed.

## 2. Materials and Methods

### 2.1. Device Design

**Figure 1:**
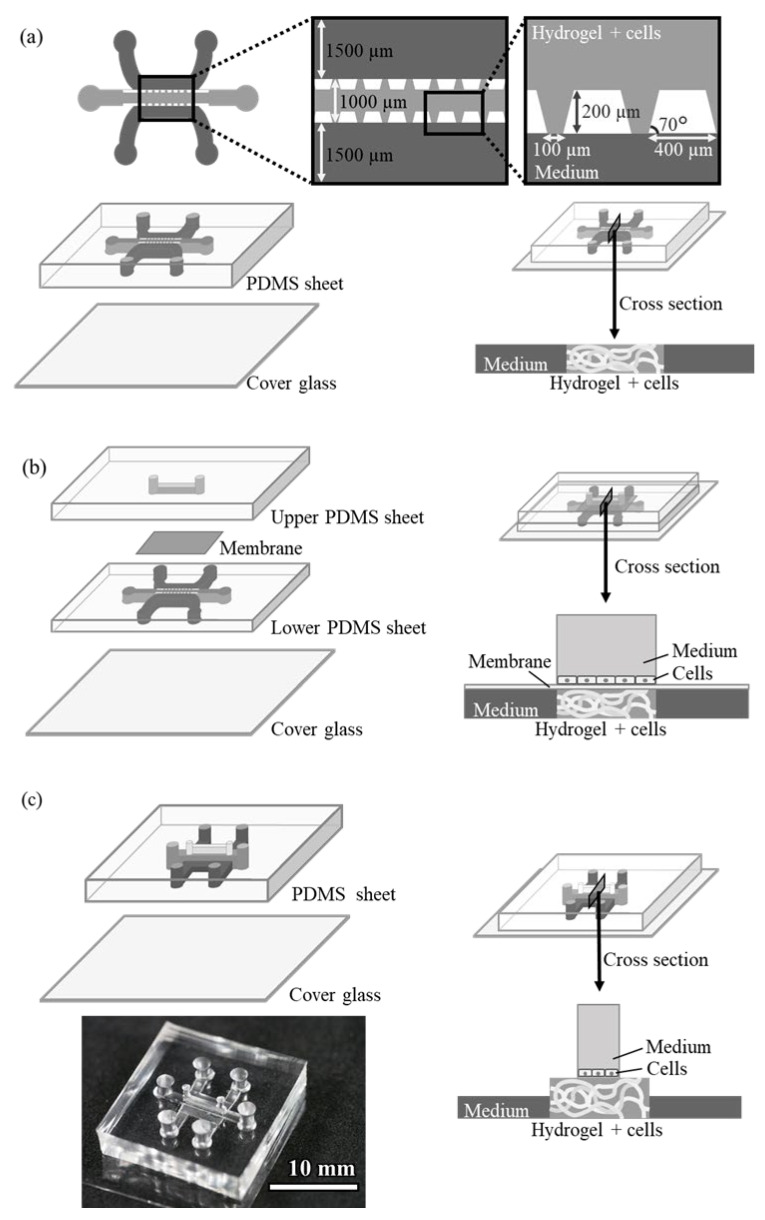
Schematic illustrations of microdevices for 3D culture with cross-sectional views. (**a**) Device A, (**b**) Device B, and (**c**) Device C.

The design of the conventional pillar array cell culture device (Device A) is shown in Figure 1a. The device consists of three structures, i.e., a central gel channel, two side medium channels, and an array of trapezoidal pillars separating them. The dimensions of the channel are 200 µm deep and 1000 and 1500 µm wide (gel and medium channels, respectively). The trapezoidal pillars separating the channels are regularly spaced 100 µm apart, and the surface tension of the pillars keeps the hydrogel-containing endothelial cells only in the gel channel. The cells in the gel grow by utilizing the medium in adjacent medium channels.

A microdevice (Device B) was designed with a third medium channel placed through a porous membrane to supply another medium to the gel channel (Figure 1b). This device has the same structure as Device A on the lower side, with another microfluidic channel installed on top of the gel channel via a polyester membrane filter with 1.0 µm pores. The upper channel can be filled with medium and another cell culture on the membrane.

The design of the device with an upper channel without a pillar array or membrane filter (Device C) is shown in Figure 1c. The channel dimensions of this device are as follows: the gel channel is 1000 µm wide or 2000 µm wide and 500 µm deep, the side medium channel is 1000 µm wide and 250 µm high, and the upper medium channel is 500 µm wide and 1000 µm high. The hydrogel in the gel channel did not leak into other channels because of the surface tension caused by the structure of the side medium channels, which were lower in height, and also by the upper medium channel, which was narrower in width than the gel channel. This structure allowed the gel to remain in the gel channel without a pillar array or membrane filter. Additionally, compared to conventional devices, obstacles at the boundary between the gel-containing cells and the culture medium are removed, making it easier for the medium to be supplied to the cells.

### 2.2. Device Fabrication

#### 2.2.1. Device A

The conventional Device A was fabricated via soft lithography using SU-8. A photoresist SU-8 3025 (Kayaku advanced materials, Westborough, MA, USA) was spin-coated on a glass slide, and a 200 µm high mold was fabricated by photolithography using a photomask. Polydimethylsiloxane (PDMS) sheets were prepared by pouring PDMS prepolymer (Silpot 184, Dow Toray, Tokyo, Japan) onto the mold and heating them at 90 °C. Inlet holes with diameters of 2 mm were punched into the resulting sheet using a biopsy punch with a plunger (BP-20F; KAI Industries, Gifu, Japan). The PDMS sheet and cover glass were irreversibly bonded via oxygen plasma treatment for 20 s in a plasma reactor (PDC200; Yamato Scientific, Tokyo, Japan). The device was sterilized by filling the channels with 70% methanol and placing them under a UV lamp for 24 h until completely volatilized before cell culture. Pipette tips that had been cut off at the tip were placed in the inlet holes of all the channels and used as medium reservoirs.

#### 2.2.2. Device B

Device B consists of two PDMS sheets, a membrane filter, and a cover glass. The same mold used for Device A was employed to fabricate the lower PDMS sheet with the lower channel. Silpot was applied on the mold at a thickness of about 200 µm and heated at 90 °C with a cover glass on top. The cured PDMS thin film was removed from the mold along with the cover glass to fabricate the lower channel. A mold of the upper channel was fabricated by attaching a 1.0 mm × 1.0 mm polystyrene strip (Evergreen Scale Models, Des Plaines, IL, USA) to an acrylic plate using plastic model cement (Tamiya, Shizuoka, Japan). A PDMS sheet with an upper channel was fabricated using this mold in the same manner as Device A. Inlet holes with diameters of 1 or 2 mm were punched at both ends of the channel using a biopsy punch. A polyester membrane filter, ipCELLCULTURE Track-etched membrane filter (pore size 1.0 µm, it4ip, Louvain-la-Neuve, Belgium), was placed between these two PDMS sheets and adhered by plasma treatment for 20 s.

#### 2.2.3. Device C

**Figure 2:**
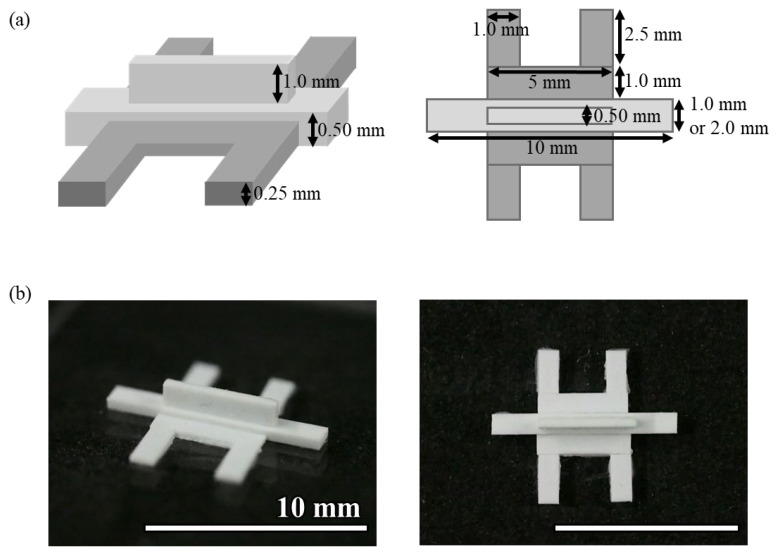
(**a**) Schematic illustrations and dimensions and (**b**) photo of the mold for Device C.

The mold for Device C was fabricated by gluing plastic strips to an acrylic plate (Figure 2). Three types of polystyrene strips of 0.50 mm × 2.0 mm, 0.50 mm × 1.0 mm, and 0.25 mm × 1.0 mm (Evergreen Scale Models) were glued to an acrylic plate using plastic model cement (Tamiya). When cutting the polystyrene strip, the cutter was used to cut it straight from directly above so that the cut surface was flat. The adhesive was applied as thinly as possible, and any excess adhesive was removed before curing. Polystyrene strips and an acrylic plate or between polystyrene strips were glued together so that there were no gaps between them. The molds were left at room temperature for at least 24 h to allow the adhesive to cure. The photolithographic molds for Devices A and B were broken after more than a dozen uses, whereas the molds made of polystyrene strips were usable more than 100 times. Because the molds were made by hand, there was a slight misalignment in the shape of the molds, but no problems were observed with hydrogel introduction or cell culture. PDMS devices were fabricated using this mold, following the same procedure as Device A. This method helps in device fabrication without the fabrication equipment required for photolithography.

### 2.3. Cell Culture

#### 2.3.1. Cells and Media

Glomerular endothelial cells were cultured to construct an endothelial network in this study. Conditionally immortalized normal human glomerular endothelial cells (GEnCs) were courtesy of Prof. Moin Saleem, University of Bristol [20]. GEnCs were cultured in the Endothelial Cell Growth Medium MV 2 (PromoCell, Heidelberg, Germany). The cells were grown at 33 °C and differentiated at 37 °C. Neonatal normal human dermal fibroblasts (NHDFs; Lonza, Basel, Switzerland), which secrete VEGF, were used to attract and elongate the GEnCs. NHDFs were cultured at 37 °C in Fibroblast Growth Medium 2 (PromoCell). Both cell types were collected by trypsin treatment after reaching confluence in a dish and used for culture in the microdevice.

#### 2.3.2. Cell Culture in a Microdevice

**Figure 3:**
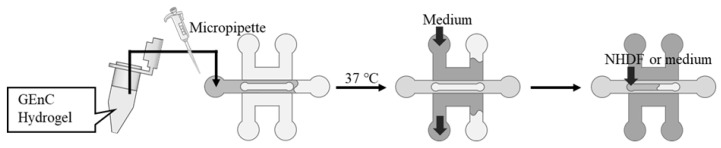
Procedure for seeding cells into Devices B and C.

The hydrogels were prepared for cell culture by mixing Matrigel growth factor-reduced basement membrane matrix (Corning, Corning, NY, USA) with fibrinogen from bovine plasma (Sigma-Aldrich, St. Louis, MO, USA) dissolved in saline at a defined concentration. The GEnCs were suspended in the hydrogel, and thrombin from bovine plasma (Sigma-Aldrich) was added and quickly filled into the gel channel using a micropipette (Figure 3). The device was incubated at 37 °C for 15 min to gel the mixed hydrogel, followed by filling up the adjacent medium channels with 400 µL of medium for GEnCs. For Devices B and C, the upper channel was filled with a medium or a medium-containing NHDF. The devices were then placed in a CO_2_ incubator at 37 °C and 5% CO_2_ to culture the cells. The culture medium in the channels was changed using a micropipette every two days.

### 2.4. Cell Observation

The cultured cells were stained with phalloidin after fixation and permeabilization to observe the cytoskeleton. The cells were washed three times with phosphate-buffered saline (PBS) after the medium was removed and subsequently fixed with 4% paraformaldehyde phosphate buffer (FUJIFILM Wako Pure Chemical, Osaka, Japan) for 30 min at room temperature. After the removal of the fixation solution, the cells were washed three times with PBS and permeabilized with 0.5% Triton X-100 (Sigma-Aldrich) in PBS for 30 min at room temperature. Subsequently, the cells were stained with 0.3% phalloidin-iFluor 555 conjugate (Cayman Chemical, Ann Arbor, MI, USA) in PBS at room temperature for 1 h. The stained cells were observed using a confocal laser scanning microscope FV-300/IX71 (Olympus, Tokyo, Japan) under the following conditions: laser intensity HV 740 V, Gain 1x, Offset 0%. Fluorescent images were acquired at 0 µm, 20 µm, 100 µm, 150 µm, and 200 µm from the bottom of the hydrogel. The total length of the cell network contained in each image was calculated using the Angiotool ver. 0.6a software [21].

## 3. Results and Discussion

### 3.1. Three-Dimensional Culture of GEnCs Using Device A

**Figure 4:**
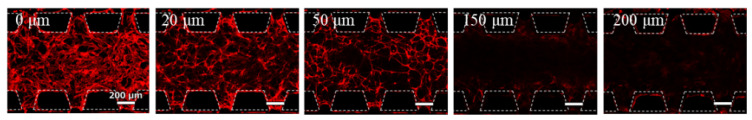
Images of phalloidin-stained GEnCs cultured in Device A taken with a confocal laser microscope. GEnCs resuspended in hydrogel were incubated for 7 days. The length shown in each picture indicates the distance from the bottom of the hydrogel.

A 3D culture of GEnCs in the hydrogel was performed using conventional Device A. Figure 4 shows the phalloidin staining images of the cells after 7 days of culture at a seeding density of 11 × 10^7^ cells/mL with 7.0 mg/mL Matrigel and 5.6 mg/mL fibrin. The spontaneous network structure of GEnCs was observed near the bottom of the gel channel and up to a position of 20 µm. However, above 50 µm from the bottom, the GEnC network decreased and no cells were present above 150 µm. The localization of the GEnCs in the lower part of the gel was likely influenced by gravity. At the 20 μm and 50 μm positions, GEnCs were observed to accumulate between the pillars. This may be due to the pillar array acting as an obstacle at the boundary between the gel and the culture medium, which may interfere with the supply of the culture medium to the cells. Therefore, it is thought that the cells in the center of the gel are deprived of nutrients and the cells have gathered more near the medium channel.

### 3.2. Three-Dimensional Culture of GEnCs Using Device B

A 3D culture of GEnCs was performed using Device B in order to attract the GEnCs to the upper part of the gel. The introduction of a medium into the upper channel is supposed to attract the GEnCs towards them, owing to the presence of VEGF in the medium. Alternatively, co-culturing NHDFs, which secrete VEGF on the membrane filter in the upper channel, is expected to attract GEnCs to the top of the gel through diffusion of VEGF. The GEnC seeding density and hydrogel composition were the same as in Section 3.1, and the NHDFs were seeded at a density of 1 × 10^4^ cells/channel.

**Figure 5:**
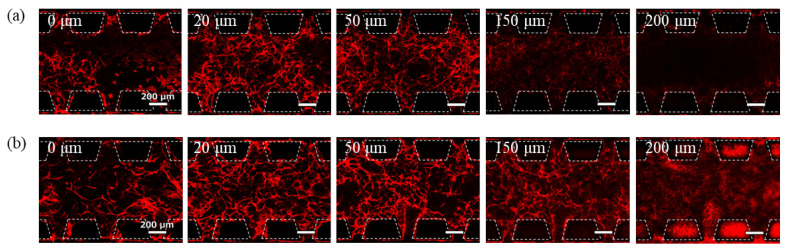
Images of phalloidin-stained GEnCs cultured in Device B taken with a confocal laser microscope. GEnCs resuspended in hydrogel were incubated for 7 days. (**a**) The upper channel was filled with medium for GEnCs. (**b**) A total of 1 × 10^4^ NHDFs were co-cultured on the membrane filter in the upper channel.

Images of the phalloidin-stained cells after 7-day culture are shown in Figure 5. Under conditions where the medium for GEnCs was introduced into the upper channel, few GEnCs were observed even at 150 µm from the bottom of the gel (Figure 5a). Although the effect of supplying the medium from the upper side of the hydrogel was confirmed, the effect was limited because the GEnC network did not spread throughout the hydrogel. Conversely, when NHDFs were co-cultured in the upper channel, the GEnC network was clearly observed even at 150 µm from the bottom of the gel (Figure 5b). Therefore, co-culture with NHDFs was more effective than supplying the medium for GEnCs in delivering VEGF from the top of the hydrogel. In the co-culture, NHDFs cultured on the membrane filter were observed at 200 µm from the bottom, and the presence of GEnCs at the 200 µm position was not confirmed.

### 3.3. Three-Dimensional Culture of GEnCs Using Device C

Device B is a culture device suitable for attracting cells to the top; however, it is difficult to fabricate, and the membrane filter interferes with cell observation. Contrastingly, Device C was designed as a simpler device that did not use a membrane and could be fabricated more easily. This device does not require photolithography during mold preparation or the lamination of the membrane filter, making it extremely easy to fabricate.

GEnCs resuspended in hydrogels were introduced into the central gel channel of Device C, and the GEnC medium was introduced into the side medium channels. Additionally, NHDFs or media for GEnCs were introduced into the upper channel of the culture. The seeding density of GEnCs and hydrogel composition were the same as described in Section 3.1, and NHDFs were seeded in the channel (1 × 10^4^ cells/channel) as described in Section 3.2.

**Figure 6:**
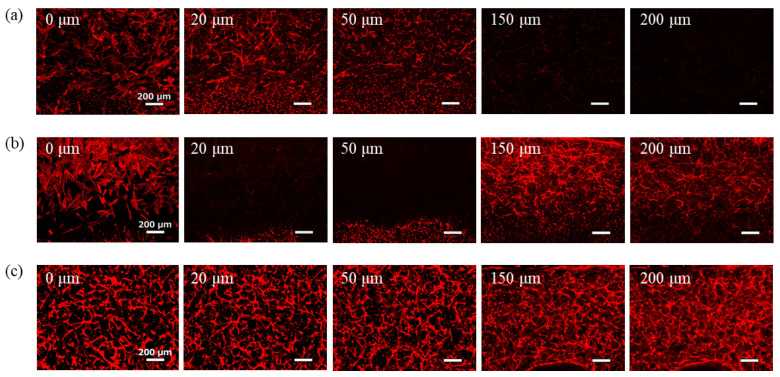
Images of phalloidin-stained GEnCs cultured in Device C taken with a confocal laser microscope. GEnCs resuspended in hydrogel were incubated for 7 days. (**a**) The upper channel was filled with medium for GEnCs. (**b**) A total of 1 × 10^4^ cells of NHDFs were co-cultured on the gel in the upper channel. (**c**) A total of 1 × 10^3^ cells of NHDFs were co-cultured on the gel in the upper channel.

Images of the phalloidin-stained cells after 7 days of culture are shown in Figure 6. When the GEnC medium was introduced into the upper channel, the effect of supplying the medium from the upper side of the hydrogel was limited (Figure 6a). In contrast, when NHDFs (1 × 10^4^ cells/channel) were co-cultured in the upper channel, most GEnCs gathered at positions higher than 150 µm, except for some cells that adhered to the glass bottom and grew in two dimensions. The GEnC network density at 20–50 µm position was significantly reduced (Figure 6b). This is thought to be due to the strong attraction of NHDFs to endothelial cells. Contrastingly, when 4.7 × 10^7^ cells/mL GEnCs were co-cultured with 1 × 10^3^ cells/channel NHDFs, good GEnC network formation was observed in all the images (Figure 6c). There was no accumulation of cells near the medium channel due to insufficient medium supply to the cells caused by the obstruction of the pillar array as observed with the conventional Device A.

Using Angiotool, an image analysis software for angiogenesis, we quantified the total length of the cell networks contained in each image (Table 1). When the distance from the bottom of the hydrogel was 0 µm, because the cells were attached to the glass surface of the channel bottom and did not form a network structure, they were omitted from the table. In the case of Device B, where the distance from the bottom was 200 µm, because NHDFs were observed on the porous membrane, it was not analyzed. In the condition where NHDFs were not co-cultured in the upper channel, the total length of the cell network decreased with increasing distance from the bottom, indicating the necessity of an upper channel in a 3D cell culture device. In Device C, where NHDFs were co-cultured at a density of 1 × 10^3^ cells/channel in the upper channel, the total length of the cell network was greater than 30 mm at all the observation positions. Therefore, we concluded that Device C was effective.

The characteristics of the three types of devices are summarized in Table 2. Device C is considered to be the most superior in all the categories: nutrient supply efficiency, device stability, manufacturing complexity, controllability, cost, and the number of media that can be supplied. In particular, the supply efficiency of the medium is extremely high because the device does not use a pillar structure or porous membrane to separate the medium channel and gel channel. In addition, the use of polystyrene rods as a mold helps to keep costs down. Furthermore, Device C is also advantageous in that it can supply three different types of culture medium, which was not possible with conventional 3D culture devices.

## 4. Conclusions

In the field of organ-on-chip development, there is an increasing need to construct tissues thicker than 200 µm with a capillary network to maintain and sustain tissue viability. When a vascular network is established within an engineered tissue, nutrients, and growth factors are transported by the flow of the culture medium, creating an in vitro model that closely resembles in vivo conditions. Microdevices for 3D tissue culture are essential for constructing vascular networks that can be incorporated into these engineered tissues. However, the construction of the device for the microvascular model reported so far requires a lot of time and money, and in order to conduct research on the microvascular model, it is necessary to prepare special equipment and acquire advanced technology for device fabrication.

In this study, we developed a mold fabrication method for microdevices of 3D culture that can be easily and inexpensively fabricated by combining plastic strips and an acrylic plate, which were used as materials for plastic model construction. This device can be fabricated easily and quickly using inexpensive and readily available materials. The widespread use of this device is expected to solve problems that have hindered the progress of 3D organ model research to date because of the limitations in fabricating or purchasing devices for tissue culture. In this study, we developed a novel Device C with an upper channel to construct thick 3D cultured tissues. By using readily available polystyrene strips, it will be possible to easily and inexpensively fabricate devices with various structures. Furthermore, by using the developed device, it will be possible to supply different culture media to cells in the gel from three directions so that this device can be used to construct various 3D organ models.

Additionally, we succeeded in constructing a 200 µm thick tissue with a GEnCs network structure by using the developed device. Human umbilical vein endothelial cells are commonly used in organ-on-chip research to study vascular network formation, but there have been no reports on the successful construction of 3D networks using glomerular endothelial cells. As organ-derived blood vessels are involved in organ-specific functions, organ-derived capillary networks should be incorporated into artificial tissues to develop organ devices with functions that are more similar to those in vivo. Particularly, the glomerular endothelial cells have a unique structure that enables efficient high-volume blood filtration during urine production. It is necessary to construct a glomerular endothelial cell-derived capillary network in order to reproduce the filtration function of kidneys in vitro. If the formation of a hollow capillary network and its function as a blood vessel can be confirmed in future studies, it is expected to make a significant contribution to the development of the kidney-on-a-chip field, the elucidation of kidney disease mechanisms, and the development of new drugs for kidney diseases.

## Figures and Tables

**Table 1:** Comparison of the differences in cell growth between the different culture conditions.

	Upper Channel	Total Length of the Cell Networks (mm)			
		Distance from the Bottom			
		20 µm	50 µm	150 µm	200 µm
Device A	-	27.0	31.8	10.6	7.6
Device B	Medium	26.2	39.6	23.1	10.6
	1 × 10^4^ NHDF cells	40.7	37.8	26.2	-
Device C	Medium	22.5	26.2	8.6	2.6
	1 × 10^4^ NHDF cells	4.3	4.9	35.0	29.2
	1 × 10^3^ NHDF cells	33.3	37.9	34.4	43.7

**Table 2:** Comparison of the differences between the three devices.

	Device A	Device B	Device C
Nutrient supply efficiency	Low	Moderate	High
Stability	High	Moderate	High
Fabrication complexity	Moderate	High	Low
Controllability	Low	Moderate	High
Cost of device fabrication	Moderate	High	Low
Number of suppliable medium	2	3	3

## Data Availability

Data are contained within the article.
